# Repeated Cytoreduction Combined with Hyperthermic Intraperitoneal Chemotherapy (HIPEC) in Selected Patients Affected by Peritoneal Metastases: Italian PSM Oncoteam Evidence

**DOI:** 10.3390/cancers15030607

**Published:** 2023-01-18

**Authors:** Enrico Maria Pasqual, Ambrogio P. Londero, Manuela Robella, Marco Tonello, Antonio Sommariva, Michele De Simone, Stefano Bacchetti, Gianluca Baiocchi, Salvatore Asero, Federico Coccolini, Franco De Cian, Marcello Guaglio, Armando Cinquegrana, Carola Cenzi, Stefano Scaringi, Antonio Macrì

**Affiliations:** 1 Advanced Surgical Oncology Center, ASUFC, DAME, University of Udine, 33100 Udine, Italy; 2 Department of Neuroscience, Rehabilitation, Ophthalmology, Genetics, Maternal and Infant Health, University of Genoa, 16132 Genova, Italy; 3 Candiolo Cancer Institute, FPO–IRCCS, 10060 Candiolo, Italy; 4 Advanced Surgical Oncology Unit, Surgical Oncology of the Esophagus and Digestive Tract, Veneto Institute of Oncology IOV-IRCCS, 35128 Padova, Italy; 5 Department of Clinical and Experimental Sciences, University of Brescia, ASST Spedali Civili, 25123 Brescia, Italy; 6 Soft Tissue U.O. Surgical Oncology-Soft Tissue Tumors, Dipartimento di Oncologia, Azienda Ospedaliera di Rilievo Nazionale e di Alta Specializzazione Garibaldi Catania, 95123 Catania, Italy; 7 General, Emergency and Trauma Surgery, Pisa University Hospital, 56100 Pisa, Italy; 8 Clinica Chirurgica 1–Ospedale S. Martino, 16132 Genova, Italy; 9 Peritoneal Surface Malignancies Unit, Fondazione Istituto Nazionale Tumori IRCCS Milano, 20133 Milano, Italy; 10 IBD Unit-DEA AOU Careggi, 50134 Firenze, Italy; 11 U.O.C.–P.S.G. con O.B.I. Azienda Ospedaliera Universitaria “G. Martino”, 98125 Messina, Italy

**Keywords:** peritoneal-surface malignancy, peritoneal metastases, hyperthermic intraperitoneal chemotherapy, cytoreductive surgery, repeated CRS-plus-HIPEC

## Abstract

**Simple Summary:**

Iterative cytoreductive surgery (CRS) and hyperthermic intraperitoneal chemotherapy (HIPEC) in patients with peritoneal metastases remain controversial regarding safety and efficacy. In this multicentric experience, we found no increased complications after the second or third procedure. Promising survival results were also observed. These data encourage iterative CRS and HIPEC, showing reassuring safety and efficacy data. It should represent a tailored treatment-strategy for selected patients.

**Abstract:**

The reiteration of surgical cytoreduction (CRS) and hyperthermic intraperitoneal chemotherapy (HIPEC) in patients affected by recurrent peritoneal metastases is still questioned regarding safety and effectiveness. This study evaluates the safety, efficacy, and associated factors of iterative CRS combined with HIPEC. This multicentric retrospective study collected data from four surgical oncology centers, on iterative HIPEC. We gathered data on patient and cancer characteristics, the peritoneal cancer index (PCI), completeness of cytoreduction (CC), postoperative complications, and overall survival (OS). In the study period, 141 CRS-plus-HIPECs were performed on 65 patients. Nine patients underwent three iterative procedures, and one underwent five. No increased incidence of complications after the second or third procedure was observed. Furthermore, operative time and hospitalization stay were significantly shorter after the second than after the first procedure (*p* < 0.05). Optimal cytoreduction was achieved in more than 90% of cases in each procedure, whether first, second, or third. A five-year (5 y) OS represented 100% of the cases of diffuse malignant-peritoneal-mesotheliomas, 81.39% of pseudomyxoma peritonei, 34.67% of colorectal cancer (CRC), and 52.50% of ovarian cancer. During the second CRS combined with HIPEC, we observed a lower rate of complete cytoreduction and a non-significantly better survival in cases with complete cytoreduction (5 y−OS CC−0 56.51% vs. 37.82%, *p* = 0.061). Concomitant hepatic-CRC-metastasis did not compromise the CRS-plus-HIPEC safety and efficacy. This multicentric experience encourages repeated CRS-plus-HIPEC, showing promising results.

## 1. Introduction

Although peritoneal metastases (PM) is an advanced disease with a poor prognosis, there is emerging evidence that cytoreductive surgery (CRS) followed by hyperthermic intraperitoneal chemotherapy (HIPEC) is a valuable treatment option in the context of multimodality management for certain patients [1,2,3,4,5]. CRS combined with HIPEC has been shown to improve long-term survival in patients with PM from appendiceal neoplasm, peritoneal mesothelioma, or PM from colorectal cancer [6,7,8,9,10]. These procedures have also been explored as a treatment option for PM from gastric, small bowel, or ovarian cancer and in primary peritoneal serous carcinoma, with encouraging results [11,12,13,14,15]. Patients with disease confined to the peritoneal cavity could be treated with CRS and HIPEC to increase survival, a major procedure with considerable morbidity and mortality [2].

Unfortunately, even after a complete or optimal first CRS and HIPEC, a high number of patients show local or systemic recurrences, determining an overall five-year survival, ranging from 11 to 19% in patients with PM from colorectal cancer and even worse outcomes in PM from gastric cancer [1,16,17].

Even if results are poor, systemic chemotherapy is the standard treatment for recurrences or progression after the first CRS and HIPEC. To improve survival, some authors have proposed repeated CRS and HIPEC, with encouraging results, but indications and outcomes are still undefined [1,2,18,19,20,21]. Furthermore, the safety of iterative procedures is challenged because of the possible complications associated with the procedure [1,4]. Indeed, the tolerability of iterative CRS and HIPEC has been questioned in the literature, due to the operation extent and the impact on a patient already compromised by an advanced-disease stage [1,4]. This study aimed to evaluate the safety, efficacy, and factors associated with the safety and effectiveness of repeated CRS and HIPEC.

## 2. Materials and Methods

### 2.1. Design, Setting, and Patients

In this retrospective chart-review, all consecutive patients who underwent CRS and HIPEC at the Unit of Surgical Oncology at the Candiolo Cancer Institute, the Veneto Institute of Oncology, the Center of Advanced Surgical Oncology in the University Hospital of Udine, or Messina University Medical School Hospital between January 1995 and May 2022, were evaluated. All centers were part of the Oncoteam for peritoneal malignancy of the Italian Society of Surgical Oncology (SICO-PSM). Among them, patients with the reiteration of CRS and HIPEC were considered. All the involved centers are high-volume and third-level institutes for peritoneal-surface-malignancies (PSM) treatment.

The selection criteria for iterative CRS and HIPEC varied between centers, but can be summarized as follows: physiological age less than 75 years; limited co-morbidity and good general status as per Eastern Cooperative Oncology Group (ECOG) performance-status score rating [22]; histological diagnosis of PM; suspected local abdominal recurrence at diagnostic imaging; unexplained weight loss associated with ascites or increment of CA125 blood values; and symptomatic partial bowel-obstruction. For patients with previous CC−1, 2, or 3 resections, the option of a second CRS was considered only in patients with a prolonged progression-free interval (>4 months), calculated starting from the first CRS and HIPEC. Exclusion criteria included the presence of extra-abdominal metastasis, poor general condition (ECOG score ≥ 3), severe vital-organ-function impairment (liver, renal, pulmonary, or cardiac function), refusal of treatment, and a low chance of achieving complete cytoreduction, based on preoperative staging. This fully anonymized chart-review assessment was executed with institutional review-board approvals. This investigation follows the Helsinki Declaration and local data-protection-authority dictates.

### 2.2. Data Collection

The authors preliminarily created a standardized data-form to collect information in every involved center. Data were retrospectively collected, focusing on clinical characteristics, the American Society of Anesthesiology (ASA) physical status [23], primary cancer histology, intraoperative staging using the peritoneal cancer index (PCI), completeness-of-cytoreduction (CC) score, postoperative complications, and survival. This study classified complications using the Clavien–Dindo classification [24]. The follow-up assessment comprised the review of clinical examinations, blood tests, pathology testing, and diagnostic imaging. All patients were usually followed up once every 3 months for a minimum of 2 years, and then once every 6 months. The same researcher checked all data forms and completed data entry to provide a uniform arrangement of data. An independent party reviewed all data.

### 2.3. Cytoreductive Surgery

All the procedures were performed using Sugarbaker’s peritonectomy technique [25]. All patients signed an informed consent preoperatively as part of the usual clinical praxis. All patients were abdominally explored, with a careful site-by-site evaluation, according to the PCI. The peritonectomy may require the following procedures: greater omentectomy with or without splenectomy; left-upper-quadrant peritonectomy; right-upper-quadrant peritonectomy; lesser omentectomy with or without cholecystectomy; pelvic peritonectomy with sleeve resection of the sigmoid colon; and eventual antrectomy [25]. In the case of PM visceral dissemination, organ resections (rectosigmoidectomy, right colectomy, total abdominal colectomy, hysterectomy, and small-bowel resection) were performed. Moreover, according to the primary tumor, appropriate resections were performed. When a small-bowel wall or mesentery localization of PM was present, removal of the cancer lesions was also achieved by electrosurgical dissection [8]. The residual disease was recorded and quantified by CC Score at the end of the surgical workup [26].

### 2.4. HIPEC

HIPEC was performed after optimal CRS and, in some cases, for palliative treatment (ascites) after incomplete CRS. HIPEC was performed intraoperatively by perfusion of a heated solution into the abdomen using the closed-abdomen technique at approximately 41.0–42.0 °C. Perfusion was carried out by means of a Performer LRT (RanD-Biotech, Medolla, MO, Italy) for a time specifically required, depending on the drug used. The chemoperfusates were the following, in association or as a single agent: mitomycin C 10 to 35 mg/m^2^; cis-diamminedichloroplatinum (CDDP) 25 to 100 mg/m^2^; doxorubicin 15 mg/m^2^; irinotecan 200 mg/m^2^; oxaliplatin 350 to 460 mg/m^2^; and 5-fluorouracil (5-FU) 400 mg/m^2^.

### 2.5. Definitions

The TNM stage was scored according to the 8th edition of the TNM classification (AJCC/UICC) [27]. The PCI, used to quantify the peritoneal spread, is a semiquantitative quantification system proposed by Paul Sugarbaker [25,28]. The PCI considers the thickness of lesion size (no macroscopic tumor, size score 0; tumor < 0.5 cm, size score 1; tumor 0.5–5 cm, size score 2; tumor > 5 cm, size score 3), and tumor distribution (abdominal-pelvic region, 0–12). The CC score used to assess the remnant disease after surgery was scored as follows: no macroscopic residual-cancer remained (CC−0); no nodule greater than 0.25 cm in diameter remained (CC−1); nodules between 0.25 cm and 2.5 cm in diameter remained (CC−2); nodules greater than 2.5 cm in diameter remained (CC−3) [26]. Optimal cytoreduction was considered to be CC−0 or CC−1 and non-optimal cytoreduction CC−2 or CC−3. Complete cytoreduction was considered to be CC−0, and incomplete cytoreduction CC−1, CC−2, or CC−3. The major postoperative complications registered were established according to grade 3 and 4 of the Clavien–Dindo criteria and up to 90 days after surgery. Postoperative death was considered as occurring within 90 postoperative days. The authors measured survival time from the date of diagnosis, the date of first CRS and HIPEC surgery, the date of second CRS and HIPEC, or the date of third CRS and HIPEC surgery to the last date of contact or death. The safety of the procedure was assessed through surgical complications, reinterventions, and postoperative death. The procedure efficacy was assessed by overall survival.

### 2.6. Data Analysis

Data were analyzed by R (version 4.2.0), considering significant *p* < 0.05. We presented data as mean (±standard deviation), median (with interquartile range–IQR), or prevalence values. In addition, 95% confidence intervals were presented when appropriate. Univariate analysis was performed using a *t*-test or Wilcoxon test in the case of continuous variables, and paired tests where appropriate. The chi-square test or Fisher’s exact test was employed for categorical variables. The Kaplan–Meier curve was drawn to show the overall survival (OS) of the selected groups. Differences between the survival in Kaplan-Meier curves were tested, employing the log-rank test. The missing values were considered missing (NA) in the statistical analyses.

## 3. Results

### 3.1. Population Characteristics

During the study period, in the four centers involved in the study, 65 patients underwent 141 procedures (CRS combined with HIPEC). Sixty-five patients underwent at least two procedures, nine underwent three, and one had five procedures. The population characteristics are illustrated in Table 1.

### 3.2. Procedure Characteristics and Complications

Table 2 shows the differences between the first and the second procedure. Intraoperative PCI was significantly lower in the second procedure than in the first procedure (*p* < 0.05). Complete cytoreduction (CC−0) was similar after the first (73.85%) and second (66.15%) procedures (*p* = 0.444). Meanwhile, the optimal cytoreduction (CC−0 or CC−1) after the first procedure was higher (98.46%) than after the second procedure (90.77%) (*p* = 0.052).

A total of 60.96% of the procedures used a combination of two drugs, and the most common combination was CCDP and mitomycin C. In 72.60% of the procedures, mitomycin C (mean dosage of 32.12 mg (±16.12)) was used, in 67.81% CCDP (mean dosage of 145.21 mg (±46.79)), in 15.22% doxorubicin (mean dosage of 44.64 mg (±15.82)), in 2.17% oxaliplatin, in 1.45% 5-FU (in CRC), and in two procedures irinotecan (in CRC and PMP).

Hepatic metastasectomy or atypical liver resection for concomitant hepatic lesions was performed in six cases: two during the first HIPEC procedure, and four during the second HIPEC procedure.

Blood transfusion, parenteral nutrition, and surgical reintervention had similar prevalence after the first and second procedures. The prevalence of grade III-IV surgical complications following the Clavien–Dindo classification was non-significantly lower after the second procedure than after the first procedure (20.00% vs. 10.77%, *p* = 0.145). However, the duration of the second procedure was significantly shorter, as well as the hospitalization stay (*p* < 0.05) (Table 2).

### 3.3. Peritoneal Cancer Index, Completeness of Cytoreduction and Procedure Safety

For a PCI value higher than 14 (the median of the distribution), no significant differences were observed in blood transfusion, parenteral nutrition, or reintervention. Moreover, severe complications (grade III-IVof the Clavien–Dindo classification) were similar (8.11% in ≤14 vs. 14.29% in >14, *p* = 0.426). Hospitalization was also similar between the two groups (13 days IQR 8.00–16.25 in ≤14 vs. 12 days IRQ 9.50–18.00 in >14, *p* = 0.802). The only significant difference was found in operative time (435 min IQR 360.00–510.00 in ≤14 vs. 570 min IQR 366.00–635.00 in >14, *p* < 0.05). Comparing complete and non-complete cytoreduction did not result in significant differences in surgical complications, reintervention, or hospitalization. Complete cytoreduction procedures were longer than non-complete ones (510 min IQR 420.00–600.00 vs. 360 min IQR 300.00–557.50, *p* < 0.05).

### 3.4. Three or More Iterative Procedures

A total of nine pseudomyxoma peritonei were treated with a third procedure, and one case had five procedures. A total of 100% of the cases had optimal cytoreduction (CC−0 or CC−1), and 87.50% had complete cytoreduction (CC−0) (Table 3). The grade III-IV Clavien–Dindo surgical complications were 11.11%, comparable to the prevalence after the first and the second procedure. In addition, the surgery duration (450 min, IQR 310–495) and the hospitalization stay (13 days, IQR 9–15) were comparable (Table 3).

### 3.5. Overall Survival

**Figure 1:**
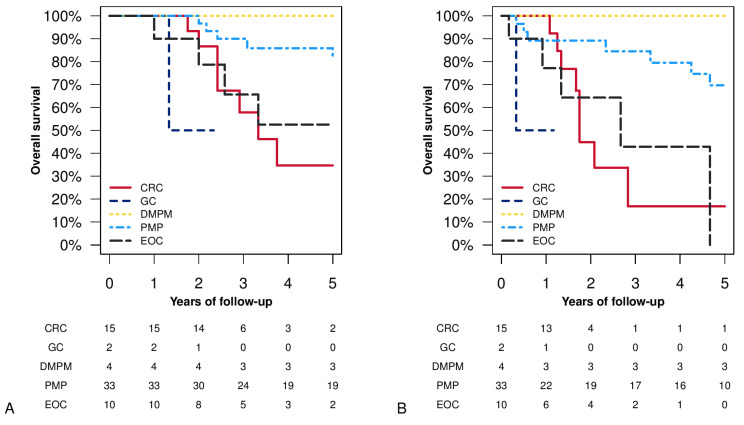
Overall survival (Kaplan–Meier analysis) stratified by histology: panel (**A**) survival from the first CRS and HIPEC procedure; panel (**B**) survival from the second CRS and HIPEC procedure. Acronyms: CRC = colorectal cancer; GC = gastric cancer; DMPM = diffuse malignant-peritoneal-mesothelioma; PMP = pseudomyxoma peritonei; EOC = epithelial ovarian cancer.

The 5-year OS from the first HIPEC of diffuse malignant-peritoneal-mesotheliomas (DMPM) was 100% (CI.95 100–100%) (all four cases treated were alive after five years) (Figure 1A). The 5-year OS of pseudomyxoma peritonei (PMP) was 81.39% (CI.95 67.72–97.81%). Meanwhile, the 5-year OS of colorectal cancer (CRC) was 34.67% (CI.95 14.46–83.13%), and of epithelial ovarian cancer (EOC) it was 52.5% (CI.95 27.22–100%) (Figure 1A). The two gastric-cancer (GC) cases had a two-year follow-up with overall survival of 50% (CI.95 12.5–100%) (Figure 1A). The only case of abdominal sarcoma resulted in death after 56 months from the first HIPEC and after 45 months from the second HIPEC. Figure 1B shows overall survival, starting from the second HIPEC.

**Figure 2:**
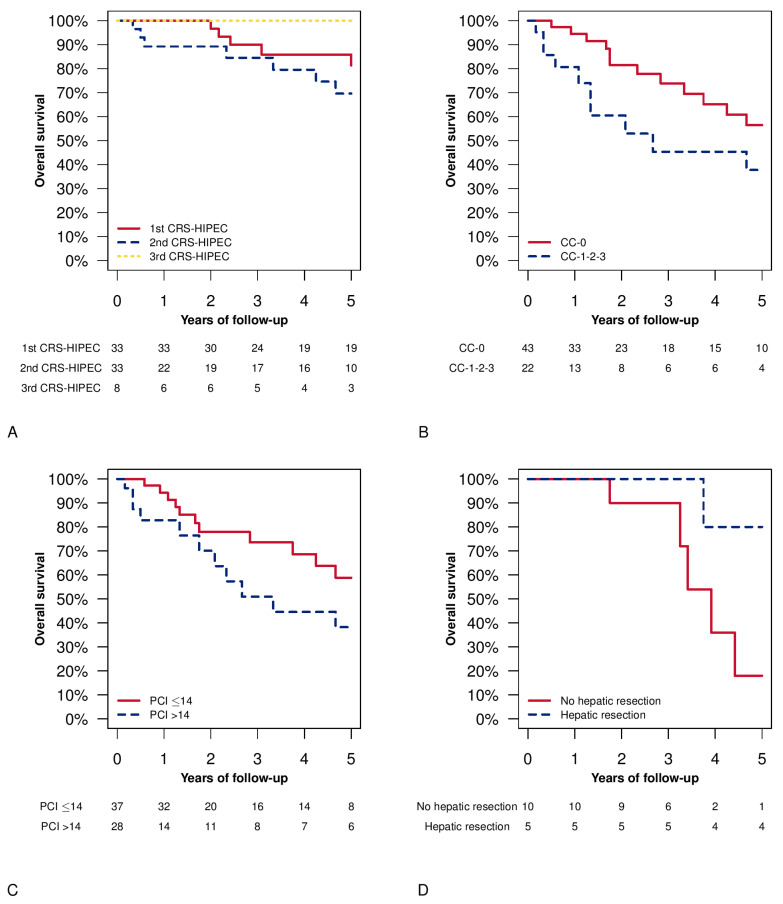
Overall survival Kaplan–Meier plots. Panel (**A**) overall survival after the first, second, and third CRS and HIPEC procedure in the patients with pseudomyxoma peritonei (PMP). Log-rank test: 1st CRS and HIPEC vs. 2nd CRS and HIPEC *p* = 0.254; 1st CRS and HIPEC vs. 3rd CRS and HIPEC *p* = 0.336; 2nd CRS and HIPEC vs. 3rd CRS and HIPEC *p* = 0.199. Panel (**B**) completeness of cytoreduction (CC) of the second procedure and overall survival from the second CRS and HIPEC (log-rank test *p* = 0.061). Panel (**C**) peritoneal cancer index (PCI) is subdivided according to the median value of the distribution at the time of the second procedure and overall survival, starting from the second CRS and HIPEC (log-rank test *p* = 0.107). Panel (**D**) overall survival from the diagnosis in patients treated during any procedure with hepatic parenchymal resection (log-rank test *p* = 0.059).

Figure 2A shows overall survival after the first, second, and third HIPEC in pseudomyxoma-peritonei patients showing similar patterns (*p* = 0.255). We observed a lower prevalence of complete cytoreduction than in the other procedures in the second procedure. Figure 2B shows the difference between complete cytoreduction and incomplete cytoreduction of the second procedure, and a non-significant better survival was observed among the complete cytoreduction group (*p*= 0.061). Furthermore, no significant differences were observed after splitting the cohort according to PCI value at the second HIPEC (higher than 14 as the cut-off). Patients with PCI lower than or equal to 14 had a 5-year OS of 58.84% (CI.95 42.11–82.22%) vs. 38.22% (CI.95 20.64–70.76%) in the PCI >14 group (*p*= 0.107) (Figure 2C).

Figure 2D shows the overall survival in patients with a concomitant (with CRS and HIPEC procedure) hepatic-CRC-metastases resection. No significant differences were observed.

The drug regimen was changed in 12 cases between the first and the second procedure. However, no significant survival differences were observed between cases treated with different drugs and cases treated with the same drug regimens as the first procedure (results for 5-year OS in the drug-change group were 45.12%, CI.95 20.95–97.17%, vs. 51.39%, CI.95 36.75–71.86% in the no-drug-change group, *p* = 0.229).

## 4. Discussion

### 4.1. Key Results

We did not find an increased incidence of complications after the second or third procedure compared with the first procedure. Furthermore, operative time and hospitalization stay were significantly shorter after the second than after the first CRS plus HIPEC. Optimal cytoreduction was achieved in more than 90% of cases in each procedure, whether first, second, or third. Moreover, the 5-year OS observed in our patients that underwent repeated procedures was 100% for DMPM, 81.39% for PMP, 34.67% for CRC, and 52.50% for patients affected by PMs from EOC, extremely similar to those seen in cases treated with a single procedure [29,30].

### 4.2. Interpretation and Comparison with The Literature

In our study and in the literature, a low prevalence of complications was found, regardless of the number of repeated CRS plus HIPEC procedures [2,5,18,19,20,21,31,32]. Moreover, in our study, an increased number of reoperations were performed after the first procedure than after the second, and even if this difference was not significant, it may be due to lower PCI in the second procedure than in the first.

In most cases, an increase in survival can be obtained after CRS and HIPEC, even if this is considered a major procedure with considerable morbidity and mortality [2]. However, PM recurrence or progression of disease following initial CRS and HIPEC are frequent: 80% of patients with colorectal cancer, 40% with peritoneal mesothelioma, and 25–44% with peritoneal pseudomyxoma find that the disease recurs within three years [18]. Recurrences localized only within the peritoneal cavity do not exceed 25% of total recurrence cases [19,21]. Further abdominal surgery, external radiotherapy, and systemic chemotherapy are the standard treatment for PM relapse, but overall survival is no longer than 12 months [21]. In particular, PM of gastrointestinal origin held a median survival of 3 months; meanwhile, that of gynecological origin showed a maximum survival of 18 months [1,33,34].

In this study, CRS and HIPEC have been proposed as offering encouraging results, even if questions about tolerability have arisen in the previous literature (prominent operation risk and impact on a patient in an advanced stage of disease and increased long-term complications) [4,18,20,35,36]. Patients who have already undergone extensive surgery and intraperitoneal perfusion typically present with a very complicated abdominal-cavity situation: adhesion and tumor infiltration divide the abdominal cavity, disrupting anatomical compartments. Therefore, complete abdominal exploration can be challenging. Still, surprisingly, all patient candidates for the procedure in our experience and in the literature were thoroughly explored after a very accurate surgical step. Moreover, following the previous literature, our data show a low proportion of reintervention, due to complications after the second procedure and patient death [2,5,18,19,20,21,31,32,37,38]. The complication rate varied among those experiences referred to in the literature. Goelse et al. found 73% of patients with complications, 40% with severe complications, and that reintervention was required in 26% of patients, in the absence of mortality [18]. Kienmanesh et al. complain that 39% of complications requiring four reinterventions, with a mortality of 2.3% [20]. Only 11.48% of our patients showed major complications, and reintervention was required in 8.20% of cases. This result is also corroborated by the previous literature, showing complications prevalence below 20% [19,20].

Despite a lower PCI in the second procedure than in the first, we registered a lower prevalence of complete cytoreduction, resulting in a lower survival among the incomplete-cytoreduction group. This finding can be partly due to the limited accuracy of presurgery imaging-techniques, often resulting in disease underestimation [39,40]. Esquivel et al. also found a decreased optimal cytoreduction in the second procedure [32]. Moreover, they emphasized the importance of an optimal cytoreduction in the second CRS with HIPEC [32]. Meanwhile, Kianmanesh et al. described an increase in the effectiveness of debulking surgery in the second rather than the first procedure [20]. Golse et al. obtained an optimal cytoreduction of 90% with a PCI of 10.3 (low tumor-burden) [18]. Meanwhile, Kianmanesh et al., in the presence of macroscopic diffuse peritoneal-disease (Gilly III-IV), obtained an optimal cytoreduction in 70% of cases [20]; and in our experience with a PCI of 14 in the second procedure, the CC−0 or 1 was obtained in 90.16% of patients.

One of the reasons for peritoneal relapse after an initial CRS and HIPEC can be the failure of chemotherapy. Therefore, Brouquet et al. changed the drug between the first and the second HIPEC, by perfusing Oxaliplatin, Mytomicin C, and Irinotecan, with no different results [19]. We changed the drug regimen in 12 cases, with similar results.

### 4.3. Strengths and Weaknesses

The main limitation of our study was the retrospective layout of the research and the absence of a proper control group. However, pairwise, we compared the first and the second procedures (CRS plus HIPEC) in the included population. Another important limitation was the small number of cases according to PSM origin, which did not allow for assessment of the factors influencing the survival of every cancer type. Another intrinsic limitation of this type of retrospective study is the possible selection bias. In this study, the PCI in the second procedure was lower than in the first. This finding could be attributed to the selection of patients who could have benefited from this type of procedure, but also to the close monitoring following the first treatment, which allowed the surgeons to proceed with a less extensive disease-burden than in the first procedure. A further limitation of the retrospective studies is the immortality bias; in this study, the survival curves were also evaluated starting from the date of the procedure, to minimize the effect of this bias on survival.

### 4.4. Relevance of The Findings, and Unanswered Questions

The results of our multicentric study add to the corpus of evidence supporting the safety of iterative procedures in PM-affected patients with the intent of improving survival for this detrimental pathology. However, many issues should still be solved before the broader adoption of this approach. Prospective studies and randomized trials are required to explore the procedure’s efficacy in different tumor histologies and drug regimens. Up until now, numerous randomized studies have confirmed the beneficial effect of cytoreduction surgery on overall survival in treating PM. According to the various randomized studies, the role of HIPEC, using well-known drugs (e.g., cisplatin or doxorubicin), is still debatable for some indications, but appears to hold a substantial role in highly selected patients with PM [41]. Promising randomized studies are currently being conducted, to assess these hypotheses [41].

## 5. Conclusions

In conclusion, our initial results encourage CRS and HIPEC repetition, resulting in safety and effectiveness. It allows patients a prolonged survival, and it should represent a tailored treatment-strategy after an adequate patient selection; further prospective multicentric studies are required.

## Figures and Tables

**Table 1:** Patient demographics.

Variable	Value
Age at diagnosis (years)	51.65 (±11.44)
BMI (kg/m^2^)	25.12 (±4.25)
Interval between diagnosis and first HIPEC (months)	6 (2–11)
Time interval between first and second intervention (months)	19 (14–29)
ASA physical Status	2 (2–2)
Center	
IDC IRCCS (Torino)	40% (26/65)
IOV IRCCS (Padova)	32.31% (21/65)
ASUFC (Udine)	21.54% (14/65)
AOU G. Martino (Messina)	6.15% (4/65)
Primary Tumor	
PMP	50.77% (33/65)
CRC	23.08% (15/65)
EOC	15.38% (10/65)
DMPM	6.15% (4/65)
GC	3.08% (2/65)
Sarcoma	1.54% (1/65)

Acronyms: BMI = body mass index; ASA = American Society of Anesthesiology; HIPEC = hyperthermic intraperitoneal chemotherapy; CRC = colorectal cancer; GC = gastric cancer; DMPM = diffuse malignant peritoneal mesothelioma; PMP = pseudomyxoma peritonei; EOC = epithelial ovarian cancer.

**Table 2:** First and second CRS and HIPEC characteristics and complications of patients who underwent at least two procedures. The *p*-values refer to the paired Wilcoxon test, chi-squared test, or Fisher’s exact test.

Variable	First CRS and HIPEC (65)	Second CRS and HIPEC (65)	*p*
Patient characteristics			
ASA	2.00 (2.00–2.50)	2.00 (2.00–3.00)	0.065
Intraoperative PCI	15.00 (11.00–27.50)	13.00 (6.00–18.50)	<0.05
Completeness of cytoreduction			
CC−0	73.85% (48/65)	66.15% (43/65)	0.444
CC−1	24.62% (16/65)	24.62% (16/65)	1.000
CC−2	0.00% (0/65)	6.15% (4/65)	0.119
CC−3	1.54% (1/65)	3.08% (2/65)	1.000
Blood transfusion	67.69% (44/65)	61.54% (40/65)	0.463
Parenteral nutrition	89.23% (58/65)	86.15% (56/65)	0.593
Reintervention	13.85% (9/65)	7.69% (5/65)	0.258
Surgical complications (Clavien–Dindo classification)			
III–IV	20.00% (13/65)	10.77% (7/65)	0.145
Operation duration (minutes)	570.00 (405.00–710.00)	480.00 (360.00–600.00)	<0.05
Hospitalization (days)	17.00 (11.00–21.00)	12.00 (8.00–17.00)	<0.05

Acronyms: ASA = American Society of Anesthesiology; HIPEC = hyperthermic intraperitoneal chemotherapy; CRS = cytoreductive surgery; CC = completeness of cytoreduction; PCI = peritoneal cancer index.

**Table 3:** Characteristics of the patients affected by PMP and treated with a third iterative CRS and HIPEC.

Variable	Value
Patients	9
ASA	2 (2–2)
Cytoreduction PCI	8.75 (±5.75)
Completeness of cytoreduction	
CC−0	87.5% (7/8)
CC−1	12.5% (1/8)
Blood transfusion	44.44% (4/9)
Parenteral nutrition	100% (9/9)
Reintervention	11.11% (1/9)
Surgical complications (Clavien–Dindo classification)	
III-IV	11.11% (1/9)
Operation duration (minutes)	450 (310–495)
Hospitalization (days)	13 (9–15)

Acronyms: ASA = American Society of Anesthesiology; HIPEC = hyperthermic intraperitoneal chemotherapy; CRS = cytoreductive surgery; CC = completeness of cytoreduction; PCI = peritoneal cancer index.

## Data Availability

The data that support the findings of this study are available, but restrictions apply to the availability of these data, which were used under license for the current study, and so are not publicly available. Data are, however, available from the authors upon reasonable request and with permission of the Internal Review Board.
